# A Treatise on Gonorrhœa and Syphilis

**Published:** 1859-11

**Authors:** 


ART. V.—*A Treatise on Gonorrhœa and Syphilis.* By SILAS DURKEE, M. D., Fellow of the Massachusetts Medical Society; Member of the Boston Society for Medical Improvement, and of the Boston Society of Natural History; Fellow of the American Academy of Arts and Sciences; Honorary Member of the Medical Society of the State of New York. With eight colored plates. Boston: John P. Jewett & Co. Cleveland, Ohio: Henry B. P. Jewett. 1859.

One would suppose that those points about which there is the greatest discrepancy of opinion in the pathology of venereal diseases would be the ones most easily settled; for example, that the causes of two of the most distinctive varieties of these maladies, propagated, as they are, by actual contact, and that only, would be as easily determined in this, as in any of the contagious maladies. On the contrary, we have had more difference of opinion among authors, and greater changes of opinion in the writings of the same authors, about this single point, than any other in the pathology of venereal disease; and more about venereal disease than any other. The importance of gonorrhœa and syphilis, both in regard to the frequency of their occurrence among all classes of society, and the terrible consequences which they entail upon some of their victims, has long been recognized; and an infallible and universal mode of treatment, if it were possible to be established in regard to any disease, would be most desirable in this. We take, up then, a new book on syphilis, with the hope and expectation of getting some light upon these vexed and important points. There are few maladies, however, which can be uniformly treated with success by the same measures. The writer in any department of medical science who inculcates such exclusive views does harm; and the practitioners who rely blindly upon him are the instruments. As the author of the work before us says in the preface, there is no "royal" plan of cure. "From time to time I have tested a great variety of remedies, and have pursued, by way of experiment, almost every line of treatment that has been brought to notice with any claims to favorable regard. But I have found no royal plan of accomplishing a speedy and certain removal of the maladies under consideration."

Dr. Durkee's work, an octavo of four hundred pages, is constituted, in great part, of an essay on "The Constitutional Treatment of Syphilis" which received the Boylston Prize in 1854. This, of course, has been modified and extended, but avowedly was the nucleus of the work now presented to us. Our first thought, in taking up a new medical work, is apt to be, whether such a work is needed by the profession. In this department there certainly exists something of a deficiency in our literature. The next inquiry is, has the work been well performed? On this point we are called upon to give our opinion. The reason this book is now presented to the profession, is explained by the author in the Preface, and is one which does infinite credit to his amiability. It is not undertaken to fill a void in the literature of the subject of which it treats; this we are led to infer, by an acknowledgment of the true reason. The prompting of an ambition to become an author are positively disclaimed; an ambition which is pardonable, at all events; especially if the individual have any genuine contribution to make to science. The real and avowed reason of its publication seems to be the *solicitation of friends.* We quote the passage in the Preface from which we make this deduction:

"In presuming to submit this work for publication, I have not been moved by the promptings of ambition to become an author. To all such aspirations I am a stranger; and were I influenced by my own inclinations, irrespective of the solicitations of others, the manuscript would have slept undisturbed."

Though an author has an undoubted right, and we, indeed, rather expect him, to give in his preface his motives in presenting a work to the profession, we do not consider the "solicitation of others" a sufficient excuse for the publication of an inferior work, nor a proper motive for writing a good one. As a personal favor, we would consider this a great deal to ask, and one in which a refusal would be excusable; as the labor of preparing an octavo of four hundred pages, to say nothing of seeing it through the press, is not inconsiderable.

In our examination of Dr. Durkee's work, and we have examined it pretty thoroughly, we do not see that it is superior to the works which are now in use, and it is certainly far inferior to the last edition of Ricord and Hunter. It seems to us carelessly thrown together; and though there is much good matter in it, we doubt if it will ever become an authority on the subject, and are confident that it cannot supersede those which have gone before.

The mechanical execution is good, though we noticed one or two typographical errors, which are the fault of the publisher. As to the illustrations, which are eight in number and colored, we think that they might be softened down with advantage; as their flaring appearance mars the beauty of the book. The space which we can give to reviews does not permit us to be very extended in this department, therefore we cannot quote in illustration of our criticism; we have, nevertheless, examined carefully, wishing to give the author full credit for all he has done. That his work has merit we do not deny; but we cannot think that its merit is sufficient to make it a successful text-book, and authority upon the subjects of which it treats.

